# The Brighton declaration: the value of non-communicable disease modelling in population health sciences

**DOI:** 10.1007/s10654-014-9978-0

**Published:** 2014-12-13

**Authors:** Laura Webber, Oliver T. Mytton, Adam D. M. Briggs, James Woodcock, Peter Scarborough, Klim McPherson, Simon Capewell

**Affiliations:** 1 UK Health Forum, Fleetbank House, 2-6 Salisbury Square, London, EC4Y 8JX UK; 2 UKCRC Centre for Diet and Activity Research, MRC Epidemiology Unit, Institute of Metabolic Science, University of Cambridge School of Medicine, Cambridge, CB2 0QQ UK; 3 British Heart Foundation Centre on Population Approaches for Non-Communicable Disease Prevention, Nuffield Department of Population Health, University of Oxford, New Richards Building, Old Road Campus, Headington, Oxford, OX3 7LF UK; 4 Nuffield Department of Obstetrics and Gynaecology, University of Oxford, John Radcliffe Hospital, Oxford, OX3 9DU UK; 5 Department of Public Health and Policy, Institute of Psychology, Health and Society, University of Liverpool, Waterhouse Building, Block B, 2nd Floor, Liverpool, L69 3BX UK

The Brighton declaration arose out of a one day workshop held in Brighton in September 2013 as part of the Society for Social Medicine annual conference. The workshop convened UK based non-communicable disease modellers to discuss the challenges and opportunities for non-communicable disease modelling in the UK. The declaration describes the value and importance of non-communicable disease modelling, both for research and for informing health policy. The declaration also describes challenges and issues for non-communicable disease modelling. The declaration has been endorsed by many non-communicable disease modellers in the UK.

## Background

Over 60 % of global deaths are attributable to non-communicable diseases (NCDs) [1]. With most developing countries experiencing a shift in disease burden away from communicable disease to NCDs, this contribution is expected to grow. To respond effectively, it will be crucial to understand these epidemics better: both how the burden of disease is anticipated to change over time based on current trends (e.g. demographic change, changes in risk factor prevalence, or changes in diseases incidence), and the effects that different interventions might have. This is important for planning health services and for developing an evidence base to inform public health policies aimed at reducing the burden of disease. While modelling is often not well understood and is frequently criticised, we argue that modelling NCDs has an important role to play in informing how society responds their increasing burden on population health.

## What is non-communicable disease modelling?

Models simplify reality—a good model represents those parts of reality that matter and leaves out those parts that do not. Modelling is the development and use of these models to understand how different inputs (e.g. behaviours) affect different outcomes (e.g. disease). NCD modelling is a method for estimating the extent to which changes in one or more risk factor (e.g. smoking and diet) affects disease and health. It has two broad uses, health impact modelling (understanding the effect of prevention, screening or treatment interventions on health) [2] and forecasting (estimating disease trends based on demographic change or predicted changes in risk factors, including making allowance for competing risk). Such modelling is sometimes extended to consider the costs to society or health systems that arise from disease, and the potential savings that may accrue from interventions. NCD models use a wide range of research methods and can answer a range of questions [3–6]. NCD modelling in the UK is performed by (and used by) a variety of institutions, academic research units, non-government organisations, as well as public bodies. Although such modelling is not new, increased availability of datasets, improved computational power and use of new methods is opening up new possibilities [7].

## The need and use for non-communicable disease modelling

Modelling is already widely used to inform decision making in both the public and private sector. Within public health it has long been recognised that the greatest influences on population health lie outside the health sector [8]. NCD modelling has, for example, been influential in the debate on minimum unit pricing [9] and is being used to integrate health into transport planning [10, 11]. NCD modelling may also be used to inform decisions on priorities for investment, for example modelling cost-effectiveness of different interventions for primary prevention of cardiovascular disease [12] or different approaches to screening [13, 14], as well as predict future trends in disease burden [13], which are important in planning health service provision.

Modelling enables us to estimate the long term and population-wide health effects of interventions. These effects are rarely observed in a single research study because of a variety of other influences on the observed outcome over time, and true randomised experiments are often not practical. While modelling studies can be cheaper and quicker than real world studies, they should be seen as a complement to such studies, and may help us get the best value out of such studies. It is often most appropriate to model when we already have a good understanding of the system being modelled, both its structures and its parameters. Often in this way modelling studies are being used to integrate evidence from different studies and different domains. Modelling can also inform empirical studies, for example to identify assumptions or key parts of evidence that may have a significant impact on model outcome (direct empirical study of these assumptions may give greater confidence in the model and its predictions). Modelling may also be used to estimate likely effect sizes to inform the size of evaluative studies.

For example our understanding of the effectiveness and cost-effectiveness of the current UK Breast Cancer Screening programme is informed by modelling [14, 15]. While estimates of the effectiveness of screening could be derived from past trials, these occurred many years ago, when incidence was lower, diagnostic technology different, and treatment options fewer. Our knowledge of the system and the parameters within the system is relatively good. It also serves as an example of modelling being complementary to, and used alongside, “empirical” evidence. While such modelling does not preclude the need for a trial, it can give a more rapid indication of where the costs and benefits lie. Indeed, estimates could be updated on a periodic basis as disease trends change and technology evolves. Further, modelling could demonstrate that the existing evidence is sufficient, such that further empirical studies may not be necessary.

## Criticisms of non-communicable disease modelling

Modelling work is often criticised. This may relate to criticisms of the underlying assumptions within the model: either the particular values (parameters) used or the model’s structure (the assumed underlying relationships or causal model). Some of these assumptions may be implicit within the model, and there is a risk that they may not be readily identified as assumptions by those building (and using) the model. This can be particularly problematic when there is either no or limited evidence to support these assumptions. Sometimes the criticism relates to the very idea of modelling, that modelling is by definition, uncertain, resulting in the view that the model will lead to unrealistic estimates. These fears may be compounded if a clear description of the model, ideally one that is accessible to a wide audience, is not given. Developing such a clear description is not always straightforward and requires the ability to articulate many of the key assumptions surrounding model structure (including implicit assumptions) to non-specialist audiences.

While criticisms of the underlying assumptions may sometimes be justified, sensitivity analyses can be used to identify which explicit assumptions are critical to the model (in terms of having a significant impact on the model outcome), and uncertainty analyses can quantify uncertainty surrounding the model’s parameters and structure; both are important for calibration and validation of the model. Within the bounds of expected values some underlying assumptions and parameters may have little effect on the model results even though the uncertainty surrounding those particular parameters may be high [16]. While there may be times that uncertainty is so great that the results cannot be used in decision making, we believe a best estimate can often be better than no estimate. Failure to model health outcomes may mean failure to consider health, alongside many of the other outcomes that may be modelled (and considered) for prospective policies, for example employment, revenues, and carbon emissions, and ill health is expensive especially in the future. It might also mean that decision making is informed by decision makers’ best guesses as to what the costs and benefits are—effectively “implicit modelling” which is unstructured, non-transparent, and not reproducible.

## Challenges for modelling

A number of challenges have been identified:

- perception that conclusions are largely assumption based and that underlying (and sometimes implicit) assumptions are being hidden or chosen in such a way to support a particular outcome;
- conceptualising and communicating issues of uncertainty, particularly to policy makers;
- communicating to both technical and non-technical audiences how the modelling was undertaken and the nature of the underlying assumptions;
- presenting study results in an accessible way to reach an audience that may not be familiar with technical details and issues of modelling;
- journals having difficulty identifying appropriate handling editors and referees with correct technical expertise;
- journal editors lacking familiarity with modelling approaches and being unsure how to appraise quality;
- findings being reported too strongly, particularly where unacknowledged or implicit assumptions may undermine the validity of the model.

## Need for standardised reporting guidelines

We recognise that some of these issues might be resolved by improved and standardised reporting of modelling studies. Transparent acknowledgements of assumptions and limitations (including using non-technical language) enables a more thorough and robust peer-review and allows readers to assess the model’s quality [17–19]. Unfortunately, to our knowledge there is no reporting guideline (e.g. Enhancing the QUAlity and Transparency Of health Research (EQUATOR) network guidelines: www.equator-network.org) for NCD modelling nor are we aware of any equivalent reporting standards.

Health economics, which often draws on modelling, has reporting guidelines and some of these cover modelling [e.g. Consolidated Health Economic Evaluation Reporting Standards (CHEERS)] [20]. There are also separate standards for the reporting of health economic modelling [21]. While these guidelines may partially address the needs of NCD modelling, we do not think they are sufficient. Besides having a large focus on economic outcomes, the economic modelling literature to which these standards apply is predominantly concerned with the cost-effectiveness of treatment of diseases on health and outcomes (for which the evidence is typically drawn from randomised controlled trials), rather than the effect of risk factor modification on health (for which the evidence may come from trials but tends to be drawn from observational studies). Moreover the health economic guidelines tend not to be used when reporting NCD modelling, particularly in the absence of an economic component to the modelling, as they are perceived as inappropriate. Consequently, we believe there is a need for specific non-communicable disease reporting guidelines within our field.

Modelling has an important role in public health and health policy. This may grow in the future, open access to data and increased computing power are facilitating the development of more sophisticated models. We recognise there is a need to improve the reporting of our work in terms of transparency and standardised reporting of both models and model results. It is also important that we continue to work in a collaborative fashion to develop the science to support modelling and capacity within the UK to undertake such work.
